# Same-day long-acting reversible contraceptive (LARC) placement: a systematic review of the benefits and barriers in the United States

**DOI:** 10.1186/s12905-026-04545-5

**Published:** 2026-05-20

**Authors:** Greta Lozano-Ortega, Andrew R. Kennedy, Simone Crespi, Vanessa Perez Patel

**Affiliations:** 1 Broadstreet HEOR, 201-343 Railway St, Vancouver, BC Canada; 2 Organon & Co, Jersey City, NJ USA

**Keywords:** LARC, Same-day long-acting reversible contraceptives, Systematic literature review

## Abstract

**Background:**

Increasing same-day placement rates of long-acting reversible contraceptives (LARCs) for individuals who are medically eligible and desire to do so is a key component of contraceptive access. The objective of this literature review was to characterize data on the effectiveness and economic impact of programs aimed at increasing same-day LARC placement in the United States (US).

**Methods:**

A systematic literature review was conducted January 2023 to identify relevant literature from 2012 onwards using Medline, EMBASE, and CINAHL databases. Studies reporting rates of same-day LARC placement, economic benefits related to specific programs or policies, or factors affecting access to same-day LARC placement were included. Key recommendations, including actionable insights to improve access to same-day LARC, were summarized.

**Results:**

Thirty-three publications, representing 31 unique studies investigating same-day LARC placement across 21 states were included. Seven studies evaluated outcomes pre- and post-implementation of programs for same-day LARC placement, and all reported an increase in the rate of same-day LARC placements post-program implementation. Two studies estimated cost savings (USD) associated with same-day LARC placement, one estimated savings of $2,117 per adolescent per year when compared to requiring a second visit, and the second estimated savings of approximately $80 million through an assumed statewide scale-up of the evaluated program. Actionable insights to promote same-day LARC placement included staff and provider training, streamlining pregnancy and screening requirements, increasing LARC availability, and providing financial assistance.

**Conclusions:**

Programs for increasing same-day LARC placement were associated with increases in same-day LARC uptake and cost savings.

## Background

In 2019, the percentage of pregnancies in the United States (US) that were unintended was 41.6% [1], a figure that is substantially higher than most other industrialized regions [1, 2]. A large proportion of those unintended pregnancies are attributable to inconsistent or incorrect use of contraception [3]. Long-acting reversible contraceptives (LARCs) such as intrauterine devices (IUDs) and subdermal implants are not user-dependent and have the lowest typical use failure rates (< 1%) compared to other forms of birth control including shorter-acting, user-dependent methods [4, 5]. However, LARC use in the US is lower than in other developed countries [6] due in part to misperceptions of efficacy, safety, and mechanism of action [7–9]. Other barriers to LARC use in the US include cost barriers, reimbursement and administrative hurdles such as insurance requirements for preapproval or step-therapy restrictions [10, 11], and patient accessibility issues [6]. 

An important barrier to accessing LARCs in the US is the common practice of requiring two visits: one for contraceptive counseling and a separate visit for LARC placement [12]. Wilkinson et al. examined the effectiveness of a two-visit protocol for placement of LARCs and reported that 32% of LARC orders were incomplete and did not lead to placement; [13] 41% were due to the patient not returning for a follow-up appointment or completing the necessary paperwork, 26% of patients declined LARC placement at follow-up, and 19% experienced insurance coverage issues [13]. 

Proponents of LARC placement on the same day as counseling argue that delaying placement increases barriers and decreases accessibility; patients may find it challenging returning for their second visit (e.g., arranging childcare, or leaving work), and may be impacted by increased out-of-pocket visit-related costs. This delay can result in patients having less effective or no contraception in the interim period, which would be misaligned with patient preference data demonstrating effectiveness as the primary driver impacting their choice of contraceptive method [14]. Consequently, as long as pregnancy can be reasonably excluded, the American College of Obstetricians and Gynecologists (ACOG) and the US Centers for Disease Control and Prevention (CDC) recommended and continue to recommend same-day LARC placement as a safe and effective practice in removing barriers to contraceptive care [15–18], following on guidance by the World Health Organization (WHO) [19–21]. 

Current evidence suggests that same-day LARC placement may increase LARC use. For example, prior to the launch of the Contraceptive CHOICE project, less than 5% of women at sentinel clinics were using LARC methods; [22] however, the removal of cost and access barriers during CHOICE led to 75% of women choosing and receiving a LARC method at their enrollment visit [22]. While individual programs have published reports, a high level review across programs is lacking. As such, this study sought to comprehensively characterize the benefits and barriers to same-day LARC placement using published data on the effectiveness and economic impact of programs for improving contraceptive access in the context of increasing same-day LARC placement.

## Methods

A systematic literature review (SLR) was conducted to identify and synthesize published evidence on the effectiveness of, and factors affecting, access to same-day LARC placement.

### Eligibility criteria, information sources, search strategies

The SLR was guided by pre-specified Population, Intervention, Comparator, Outcomes, and Study design (PICOS) criteria (Appendix A in Supplementary Material) and followed the Preferred Reporting Items for Systematic reviews and Meta-Analyses (PRISMA) guidelines. Studies of women and adolescent girls of reproductive potential in the US who were seeking, using, or eligible to use a LARC were included. Studies focusing on healthcare providers (e.g., primary care physician, manager of a multi-specialty clinic), representatives of healthcare organizations (e.g., independent primary care practices, hospitals, integrated delivery networks) and policy makers (e.g., health plan administrators, Medicare beneficiaries, state Medicaid directors) were also included to inform effectiveness estimates and to provide insights for successful LARC placement program implementation.

A literature search was executed using MEDLINE, Embase, and CINAHL to identify studies published in English between 1/January/2012 to 6/January/2023. The search strategy was developed using terms related to the population, study design, and outcomes of interest (Appendix B in Supplementary Material). Search results were imported into EndNote (EndNote X8, Clarivate Analytics); duplicate articles were removed following the algorithm established by Bramer et al. [23]

For inclusion, studies must have reported rates, rate ratios, and/or odds ratios of same-day LARC placement relating to specific programs or policies; authors’ perceived barriers or facilitators to same-day LARC placement; or the economic impact of implementing a policy or program aimed at increasing same-day LARC placement. All observational quantitative, qualitative, or mixed-methods studies, and randomized or non-randomized controlled trials were included. Abstract and full text screening were conducted by two independent reviewers according to PRISMA guidelines [24]. 

### Data extraction

Independent double data extraction was performed using a customized Microsoft Excel workbook. Extracted data included details on study design and methodology, baseline demographic and clinical characteristics of the included user populations, details of implemented LARC programs, and outcomes of interest.

For dichotomous and categorical variables, counts and proportions were extracted. Means/medians with standard deviations/interquartile ranges were extracted for continuous variables. Estimates of effect of the implemented program and associated 95% confidence intervals (CIs) were extracted whenever reported. Discrepancies between the extracted data were resolved through discussion to achieve consensus.

Authors’ statements related to perceived barriers to, or facilitators of, same-day LARC placement were extracted into free text fields.

### Synthesis

From the set of eligible studies, study and patient characteristics were tabulated, including study methodology, type of data source, patient/provider sample size, and type of LARC device investigated. Details on patients’ age, race/ethnicity, and insurance coverage were documented. Subgroups were captured as originally presented, and details captured on US state, clinic type and location, and/or type of contraceptive care received, where applicable.

Details of LARC placement programs were synthesized and outcomes from pre- and post-program implementation were tabulated and compared visually, where available, using effect measures as reported (e.g., percentages, odds ratios [ORs]). Program- and provider-relevant effectiveness outcomes included the frequency of placing LARC in a single visit and/or the number of devices they placed. For patients, program effectiveness was measured by the receipt of a LARC device during the initial visit.

Features implemented across LARC programs to aid with same-day LARC uptake were synthesized into major categories; the most frequent features were narratively summarized.

Conclusions and recommendations made by the authors of the included studies regarding access to same-day LARC placement along with author-identified barriers and facilitators were narratively summarized. These recommendations were synthesized and characterized into key domains of healthcare access. Actionable insights were then derived through this synthesis. Economic outcomes were extracted and the impact of same-day LARC placement on cost savings was narratively summarized.

Quality assessment was undertaken by a single reviewer using the Mixed Methods Appraisal Tool (MMAT) for all included studies (Appendix C in Supplementary Material) [25]. These assessments were then independently validated by a second reviewer.

## Results

**Fig. 1:**
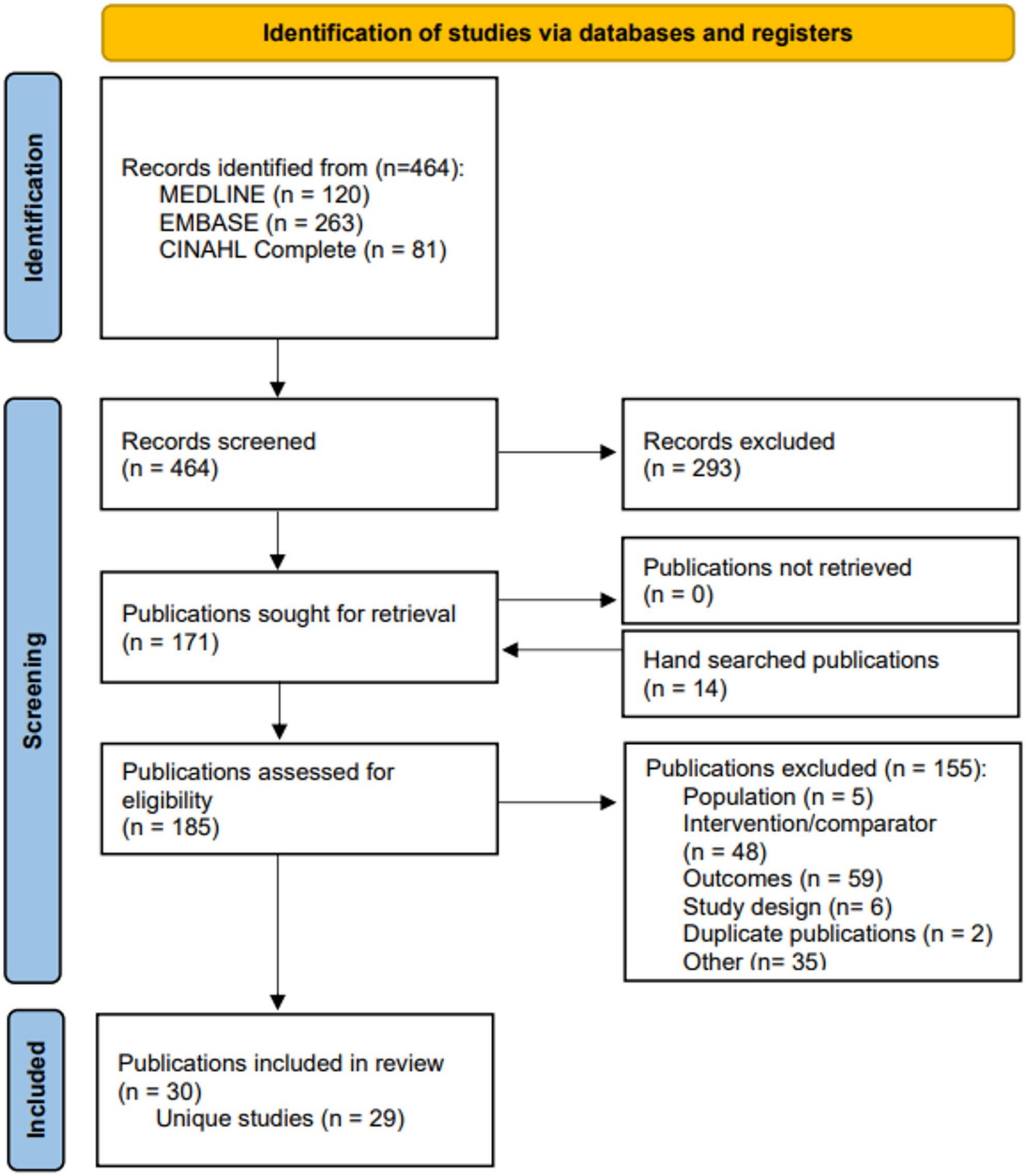
PRISMA flow diagram

A total of 30 full-text publications were included in this review, representing 29 unique studies (Fig. 1). Two publications were extensions of their respective original studies and reported on the same initiative and population, however, the subsequent publications reported additional relevant information and/or longitudinal data [26–29]. Seventeen studies described outcomes among individuals receiving same-day LARC within 15 unique programs, 2 studies described the economic impact of same-day LARC, one study characterized immediate post-surgical abortion LARC uptake, and all 29 studies were used to identify and synthesize actionable insights.

### Study characteristics

Included publications utilized a variety of designs and investigated same-day LARC placement across 21 states (Table 1). Four publications employed a mixed-method design [29–32], two used qualitative methods [33, 34], and the remaining 23 used quantitative designs [26–28, 35–55]. Among the quantitative publications, most were observational [26–28, 36–45, 47–54], one was a non-randomized controlled time-trend trial [35], one performed a cost-minimization analysis [55], and another conducted a cost-effectiveness analysis of the CHOICE project [46]. Twenty-one publications used primary data sources (e.g., interviews or surveys) [26–31, 33–35, 39–45, 48, 49, 51, 53, 54], seven used secondary data from medical claims and electronic medical records (EMR) [36–38, 46, 47, 50, 52], and one used both primary and secondary data [32]. 

**Table 1:** Study characteristics

Author, year		Program name	Study setting			Study design				Device
			Geographic region	Coverage	Multi-center	Type	Data source	Year(s) of study	Overall Sample size	
Provider										
Reeves, 2023		-	USA	Both urban and rural	Yes	Quantitative	Provider survey	2019	1063	IUD
Cohen, 2022		Family Planning Elevated Contraceptive Access Program (FPE CAP)	Utah	Both urban and rural	Yes	Mixed	Provider checklists, video recordings and programmatic monitoring data.	2020	15	IUD
Vohra-Gupta, 2022		Healthy Texas Women (HTW), Title X, Family Planning Program (FPP)	Texas	-	Yes	Quantitative	Provider survey	2016–2017	114	IUD and Implant
Song, 2022		-	Illinois	Both urban and rural	Yes	Qualitative	Provider interview	2020	40	IUD and Implant
Lim, 2020		-	Pittsburgh	-	Yes	Quantitative	Provider survey (secret-shopper)	NR	50	Hormonal IUD
Davis, 2020		Buy and Bill	Hawai’i	Urban	No	Quantitative	Claims	2016–2017	37	IUD and Implant
Judge-Golden, 2020		-	Western Pennsylvania	Both urban and rural	Yes	Quantitative	Provider survey and CDC selected practice recommendation for contraceptive use	2018–2019	153	IUD and Implant
Serpico, 2020		-	Ohio	Both urban and rural	Yes	Quantitative	Provider audit (secret shopper)	NR	340	IUD
Tepper, 2020		Zika Contraceptive Access Network (Z-CAN)	Puerto Rico	Both urban and rural	Yes	Quantitative	Provider survey	2017	116	IUD and Implant
Harper, 2020		Contraceptive training intervention	USA	Both urban and rural	Yes	Quantitative	Provider survey	2013–2019	3557	IUD and Implant
Katz-Wise, 2020		-	New England	Both urban and rural	Yes	Qualitative	Provider interview	NR	28	IUD and Implant
Natavio, 2018		California Family PACT (Planning, Access, Care, and Treatment)	California	-	Yes	Quantitative	Provider survey (secret-shopper)	2016	284	IUD and Implant
Loyola Briceno, 2017		Performance Measure Learning Collaborative (PMLC)	USA	Both urban and rural	Yes	Quantitative	Provider self- assessment	2015–2016	12	IUD and Implant
Kelly, 2017		-	USA	Both urban and rural	Yes	Quantitative	Provider survey	2015	390	IUD and Implant
Romero, 2017		National demonstration project providing technical assistance and training (TTA)	Alabama, Connecticut, Georgia, Massachusetts, New York, North Carolina, Pennsylvania, South Carolina, and Texas	-	Yes	Mixed	Provider assessment	2012–2013	48	IUD and Implant
Biggs, 2015		California Family PACT (Planning, Access, Care, and Treatment)	California	Both urban and rural	Yes	Quantitative	Provider survey	2011	636	IUD and Implant
Romero, 2015*		National demonstration project providing technical assistance and training (TTA)	Alabama, Connecticut, Georgia, Massachusetts, New York, North Carolina, Pennsylvania, South Carolina, and Texas	-	Yes	Quantitative	Provider assessment	2011	51 health centers and 48,850 adolescent clients	IUD and Implant
Luchowski, 2014		-	USA	-	Yes	Quantitative	Provider survey	NR	1221	IUD and Implant
Biggs, 2013		Statewide Initiatives	Iowa and Colorado	-	Yes	Mixed	Provider survey	2012	45	IUD and Implant
User										
Stuart GS, 2023		-	North Carolina	-	Yes	Quantitative	Electronic medical records	2019–2021	4,599	IUD and Implant
McColl, 2022		Delaware Contraceptive Access Now (DelCAN)	Delaware and Maryland	Both urban and rural	Yes	Quantitative	Electronic medical records	2012–2019	843, 211	IUD and Implant
Castaño, 2020		-	New York	Urban	Yes	Quantitative	Electronic medical records	2009–2012	592	Hormonal IUD
O’Laughlin, 2020		Pregnancy Reasonably Excluded Guide (PREG) Evaluation	Minnesota	Urban	No	Quantitative	User survey	2015–2018	981	IUD and Implant
Buckel, 2019		Enhanced Care and Complete CHOICE	Midwest (Missouri and Tennessee)	Urban	Yes	Quantitative	User survey	2014–2015	1008	IUD and Implant
Landgraf, 2019^†^		Quality improvement project	Colorado	-	Yes	Mixed	Chart review and survey	NR	40	IUD and Implant
Roe, 2019		-	Massachusetts	-	Yes	Quantitative	Electronic medical records	2012–2017	26,858	IUD and Implant
Wilkinson, 2019		-	Indiana	-	Yes	Quantitative	Cost minimization analysis	2017–2018	--	IUD and Implant
DeBoer, 2018		Quality improvement project	Georgia	Rural	No	Quantitative	Chart review	2016–2017	15	IUD and Implant
Lathrop, 2018		Zika Contraceptive Access Network (Z-CAN)	Puerto Rico	Both	Yes	Quantitative	Survey	2016–2017	21,124	IUD and Implant
Madden, 2018		CHOICE Project	St Louis	Urban	Yes	Quantitative	Chart review + EMR	2007–2013	5061	IUD and Implant

*Abbreviations*: *CAP* Contraceptive Access Program, *CDC* Centers for Disease Control and Prevention, *DelCAN* Delaware Contraceptive Access Now, *FPE* Family Planning Elevated, *FPP* Family Planning program, *HTW* Healthy Texas Women, *IMPACCT* Innovative Model of PAtient-Centered ContracepTion, *IUD* intrauterine device, *NR* Not reported, *PACT* Planning, Access, Care, and Treatment, *PMLC* Performance Measure Learning Collaborative, *PREG* Pregnancy Reasonably Excluded Guide, *TTA* Technical assistance and training, *USA* United States of America, *Z-CAN* Zika Contraceptive Access Network

*Romero, 2015 reports on the same population as Romero, 2017

^†^These studies also reported on provider populations, however, the study focus was on the user population

### Study samples

Nineteen studies included healthcare provider samples [26–34, 37, 39–41, 43–45, 49, 51, 53, 54] and eleven focused on samples of reproductive-aged women seeking contraceptive care in the US [32, 35, 36, 38, 42, 46–48, 50, 52, 55], of which one also included providers [32]. Sample sizes of reproductive-aged women ranged from 15 to 35,847 across studies, with most (83%) studies including adolescents (Table 2). The percentage of women *≤* 20 years of age varied across the studies from < 10% [32] to 100% [50]. Where reported, mean ages were primarily in the 25–29 year range, with one study reporting a mean age of 30.6 years [48]. Insurance coverage type was also reported in over half the studies [35, 38, 42, 46, 47, 50, 52]. Public insurance represented the majority of coverage in four studies [35, 42, 47, 50], commercial/private insurance in one study [52], and patients paying out-of-pocket (self-pay/uninsured) in two studies [38, 46]. 

**Table 2:** Patient characteristics, for studies that included LARC users

Author, year	Description of population	Number enrolled (*N*)	Age (years)	Race/Ethnicity					Insurance		
			Mean (SD)	Black, *n* (%)	White, *n* (%)	Asian, *n* (%)	Hispanic / Latino, *n* (%)	Other, *n* (%)^*^	Commercial / Private, *n* (%)	Public, *n* (%)	Self-pay / Uninsured, *n* (%)
Stuart GS, 2023	Outpatients for IUD/ contraceptive implant visiting Obstetrics and Gynecology physician	3,411	-	618 (18.1)	2114 (62.0)	68 (2.0)	381 (11.2)	611 (17.9)	2210 (64.8)	1083 (31.8)	118 (3.5)
	Outpatients for IUD/ contraceptive implant visiting Family Medicine physician	996	-	208 (20.9)	130 (13.1)	36 (3.6)	125 (12.6)	622 (62.5)	700 (70.3)	206 (20.7)	90 (9.0)
	Outpatients for IUD/ contraceptive implant visiting Internal Medicine physician	112	-	12 (10.7)	80 (71.4)	2 (1.8)	9 (8.0)	18 (16.1)	106 (94.6)	4 (3.6)	2 (1.8)
	Outpatients for IUD/ contraceptive implant visiting pediatric physician	80	-	19 (23.8)	39 (48.8)	1 (1.3)	19 (23.8)	21 (26.3)	33 (41.3)	45 (56.3)	2 (2.5)
McColl, 2022	Patients receiving LARC at a DelCAN participating agency	6,676	-	2643 (39.6)	2997 (44.9)	-	914 (13.7)	120 (1.8)	-	6676 (100)	-
	Patients receiving LARC at a non-participating Delaware agency	688	-	233 (34)	346 (50.3)	-	87 (12.7)	21 (3.1)	-	688 (100)	-
	Patients receiving LARC in Maryland	35,847	-	17,565 (49)	11,972 (33.4)	-	2867 (8)	3441 (9.6)	-	35,847 (100)	-
Castaño, 2020	Women who received same day IUD and met the pregnancy checklist criteria	353	26.1 (7.1)	21 (5.95)	48 (13.6)	-	219 (62.04)	65 (18.41)	-	-	-
	Women who received same day IUD and did not meet the pregnancy checklist criteria	239	26.7 (7.4)	15 (6.28)	44 (18.41)	-	128 (53.56)	52 (21.76)	-	-	-
O’Laughlin, 2020	Women aged 18 to 50 who were seen in a primary care gynecology clinic after PREG implementation	1,012	30.6 (8.6)	33 (3.3)	878 (86.8)	47 (4.6)	36 (3.6)	54 (5.3)	-	-	-
Wilkinson, 2019	Decision model from the perspective of Indiana State Medicaid. Base case was a 16-year-old patient presenting for care and desiring LARC.	-	-	-	-	-	-	-	-	-	-
Buckel, 2019	Enhanced care	502	25.3 (6.5)	374 (74.5)	96 (19.1)	-	39 (7.8)	32 (6.4)	70 (13.9)	311 (62)	121 (24.1)^†^
	Complete CHOICE	506	26.8 (7.4)	320 (63.2)	159 (31.4)	-	66 (13)	27 (5.3)	82 (16.2)	274 (54.2)	150 (29.6)^†^
Landgraf, 2019	Adolescent patients who sought SRH	40	-	-	-	-	-	-	-	-	-
Roe, 2019	Patients with immediate post-abortion IUD uptake	4,941	-	780 (15.8)	2378 (48.1)	202 (4.1)	933 (18.9)	1581 (32)	1609 (32.6)	3045 (61.6)	287 (5.8)
	Patients with immediate post-abortion Implant uptake	1,885	-	378 (20)	769 (40.8)	52 (2.8)	480 (25.5)	686 (36.4)	441 (23.4)	1356 (71.9)	88 (4.7)
	Patients who did not initiate immediate post-abortion IUD	21,917	-	3698 (16.9)	10,013 (45.7)	1393 (6.3)	3431 (15.7)	6813 (31.1)	5960 (27.2)	10,377 (47.3)	5580 (25.5)
	Patients who did not initiate immediate post-abortion Implant	24,973	-	4100 (16.4)	11,622 (46.6)	1543 (6.2)	3884 (15.6)	7708 (30.8)	7128 (28.6)	12,066 (48.3)	5779 (23.1)
DeBoer, 2018	Women who had a LARC placed based on the 2007 criteria	70	-	-	-	-	-	-	-	-	-
	Women who had a LARC placed based on the 2017 criteria	15	-	-	-	-	-	-	-	-	15 (100)
Lathrop, 2018	Women of reproductive age who participated in Z-CAN	21,124	-	-	-	-	-	-	8813 (42)	10,786 (51)	1111 (5)
Madden, 2018	Contraceptive CHOICE Project participants who were current Missouri Medicaid beneficiaries or were uninsured and reported household incomes < 201% of the FPL	5,061	-	3,072 (60.7)	1,594 (31.5)	-	323 (6.4)	389 (7.7)	-	1,442 (28.5)	3,618 (71.5)
	A simulated comparison group of women who were receiving care through the Missouri Title X program	404,352	-	118,475 (29.3)	276,172 (68.3)	-	22,643 (5.6)	10,108 (2.5)	-	109,983 (27.2)	294,368 (72.8)

*Abbreviations*: *FPL* Federal poverty line, *IUD* Intrauterine device, *LARC* Long-acting reversible contraception, *PREG* Pregnancy Reasonably Excluded Guide, *SRH* Sexual reproductive health, *Z-CAN* Zika Contraceptive Access Now

*Other can include American Indian / Alaskan Native, Native Hawaiian, Pacific Islander undeclared, or unknown

†One patient in this group had unknown insurance

### Effectiveness of same-day LARC placement programs

Fifteen programs aimed at increasing same-day LARC uptake were captured in 18 publications (Appendix Table C1 in Supplementary Material) [26–32, 35, 37–39, 42, 44, 46–48, 53, 54], with various program features attributed by the investigators to be effective at improving access to same-day LARC placement. Five categories of program features were highlighted across publications, with multiple features being implemented in some programs (Table 4): cost support (11 programs) which included the implementation of practices such as effective reimbursement, no-cost contraception, and cash grants for clinics; [26, 27, 30, 31, 35, 37, 42, 44, 46, 47, 53, 54] provider training/education (6 programs); [28, 29, 31, 39, 42, 47, 53] contraceptive use best practices (5 programs); [28, 29, 32, 38, 44, 48] patient-centered counseling (5 programs); [35, 42, 44, 46, 53] and increase in on-site availability (3 programs) [42, 44, 47, 53]. Best practices for contraceptive use included in the programs are recommendations outlined by ACOG [4] and the CDC [56] and include practices such as quick-starting contraceptives, removing STI testing and results requirements, and the PREG checklist. Programs in Table 3 are organized based on type of program features, with programs potentially requiring the largest monetary resources first, followed by those requiring less financial resources.

**Table 3:**
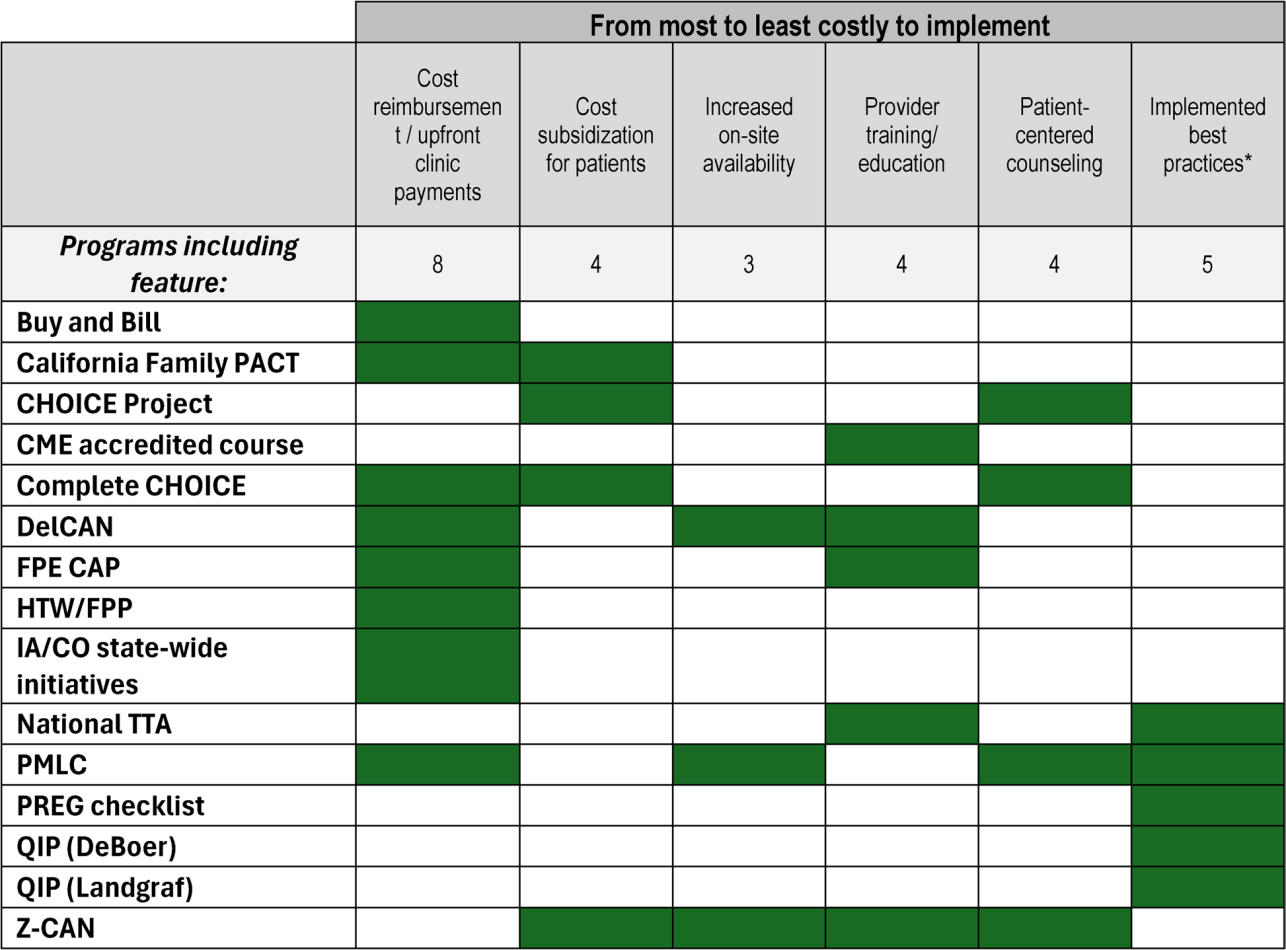
Implemented program features and resulting key takeaways

*Abbreviations*: *CAP* Contraceptive Access Program, *CME* Continuing Medical Education, *CO* Colorado, *DelCAN* Delaware Contraceptive Access Now, *FPE* Family Planning Elevated, *FPP* Family Planning program, *HTW* Healthy Texas Women, *IA* Iowa, *IMPACCT* Innovative Model of PAtient-Centered ContracepTion, *IUD* Intrauterine device, *LARC* Long-acting reversible contraception, *PACT* Planning, Access, Care, and Treatment, *PMLC* Performance Measure Learning Collaborative, *PREG* Pregnancy Reasonably Excluded Guide, *QIP* Quality Improvement Project, *TTA* Technical assistance and training, *Z-CAN* Zika Contraceptive Access Network

*Contraception implementation best practices are also available from the CDC (18)

Note: Total numbers do not add up to 19 due to some programs utilizing multiple features to improve LARC access

**Fig. 2:**
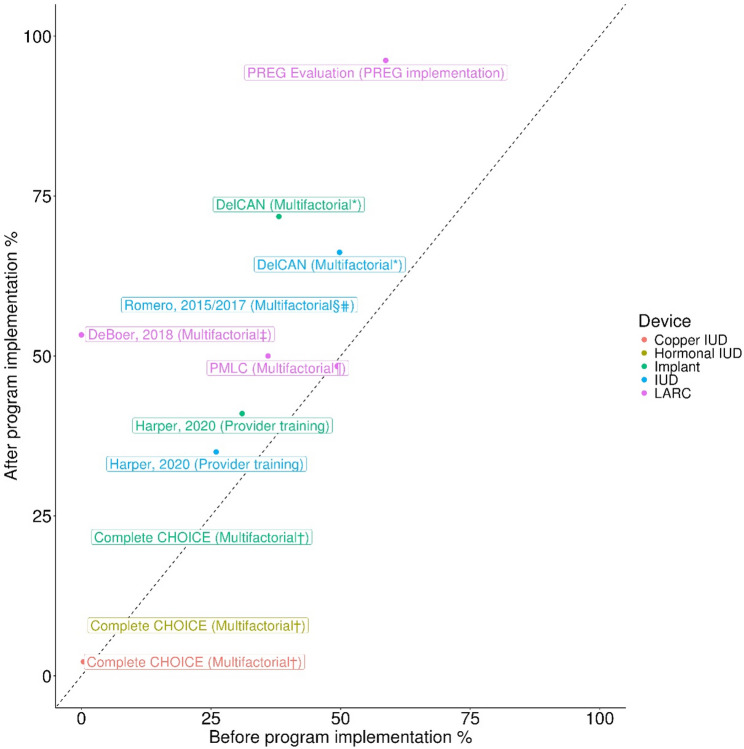
Before vs. after rates of same-day LARC placement. Abbreviations: IUD: intrauterine device; LARC: long-acting reversible contraception ^*^: Financial / stocking support, staff and physician education and training, admin support, and participation incentives ^†^: Patient counseling; healthcare practitioner education and training, and financial support ^‡^: Updated criteria for same-day placement (pregnancy assessment checklist, same-day STI testing for high-risk women only, cervical cancer screening not required) ^¶^: Implementation of systems for same-day provision of all contraceptive methods ^§^: Implementation of evidence-based clinical practices to support provision of youth-friendly reproductive healthcare; change in same-day placement from year 1 to year 2 of program ^#^: Year 1 to Year 3 data

Nine programs reported quantitative outcomes related to same-day LARC placements before and after program implementation (Table 4) [28, 29, 35, 38, 39, 44, 47, 48]. Eight saw an increase in same-day LARC uptake after program implementation; increases ranged from 2% to 53% across programs, with improvement in rates post-program consistently observed across program types (Fig. 2). Five evaluated multifactorial programs; these programs varied with respect to the elements implemented and incorporated changes related to financial and LARC device stocking support, healthcare provider training, patient counseling, and/or removal for testing requirements among low-risk patients (e.g., testing for pregnancy or for sexually transmitted infection [STI]). Over 5 years of the Delaware Contraceptive Access Now (DelCAN) program, rates of same-day IUD placements rose from 49.8% to 66.2% while same-day implant placements rose from 38.1% to 71.8% (program features included cost support, provider training, and increased on-site availability) [47]. Non-participating comparison clinics in Delaware (IUD) and Maryland (IUD and implant) observed declines in same-day placement rates over the same period. Implementation of the Complete CHOICE program (features include cost support and patient-centered counseling) showed higher rates of same-day placements of hormonal IUDs (7.9%), copper IUDs (2.2%), and implants (21.7%) as compared to the comparison Enhanced care control group (2.0%, 0.4%, and 3.4% respectively) [35]. 

One of the largest reported increases in number of same-day LARC placements was solely focused on the removal of human chorionic gonadotropin (hCG) pregnancy testing among patients with low risk of pregnancy (58.7% same-day LARC placements before vs. 96.2% after program implementation; Table 4). One program focused on healthcare provider training and saw a statistically significant increase in the number of same day placements (Table 4; odds ratio, 1.6 [95% CI: 1.4, 1.8] for IUD and 1.5 [1.3, 1.7] for implants) [39]. Another training-focused program observed increases across multiple provider knowledge domains, including 61% of providers reporting confidence in knowing who is eligible for no-cost contraception through the Family Planning Elevated (FPE) contraceptive access program, compared to 38% prior to training [31]. The California Family PACT program observed the only decrease in availability of same-day placement of LARCs among the included clinics, which the authors attributed primarily to an overwhelming non-provision on site of LARC overall, as well as ongoing policy and practice issues that inhibited adoption of same-day placement [26, 27]. In 2011, a survey of clinic staff reported 42% and 53% of providers offered IUDs and implants respectively, with only 1 visit required; this dropped to 16% confirming same-day LARC placement, with another 14% noting conditional same-day placement.

**Table 4:** Programs implemented in the United States to improve access to same-day LARC placement and their effect

Author, year	Program name	Study group	Device	Outcome definition	Control / before program implementation			Intervention / after program implementation			Comparison analysis	Effect size (95% CI)
					Sample size (*N*)	Time period / control	*n* (%)	Sample size (*N*)	Time period / control	*n* (%)		
McColl, 2022	Delaware Contraceptive Access Now (DelCAN)	DelCAN participating clinics	IUD	Same-day placement of LARC	6,676	2014	3,322 (49.8)	6,676	2019	4,418 (66.2)	DID (Race and age adj.)	25.6 (15.1–36.1)
		DelCAN participating clinics	Implant	Same-day placement of LARC	6,676	2014	2,543 (38.1)	6,676	2019	4,795 (71.8)	DID (Race and age adj.)	17.4 (9.2–25.6)
	-	Delaware non-participating clinics	IUD	Same-day placement of LARC	688	2014	424 (61.6)	688	2019	396 (57.6)	-	-
		Delaware non-participating clinics	Implant	Same-day placement of LARC	688	2014	342 (49.7)	688	2019	437 (63.5)	-	-
	-	Maryland comparison clinics	IUD	Same-day placement of LARC	35,847	2014	19,956 (55.7)	35,847	2019	18,207 (50.8)	-	-
		Maryland comparison clinics	Implant	Same-day placement of LARC	35,847	2014	22,071 (61.6)	35,847	2019	19,264 (53.7)	-	-
Cohen, 2022	FPE CAP	Clinic staff who participated in the training intervention	LARC	Participants who responded “Strongly agree” to: I know who is and is not eligible for no-cost contraception through FPE	71	Pre-training simulation	27 (38.0)	71	Post-training simulation	43 (60.6)	-	-
				Participants who responded “Strongly agree” to: I have a good understanding of how FPE works within my clinic	71	Pre-training simulation	27 (38.0)	71	Post-training simulation	46 (64.8)	-	-
				Participants who responded “Strongly agree” to: My clinic can easily provide all methods of reversible contraception to everyone who wants them	71	Pre-training simulation	33 (46.8)	71	Post-training simulation	45 (63.4)	-	-
				Participants who responded “Strongly agree” to: I am familiar with the challenges that clients can experience when seeking contraception services	71	Pre-training simulation	31 (43.7)	71	Post-training simulation	54 (76.6)	-	-
				Participants who responded “Strongly agree” to: I can help a client get any contraceptive method they want	71	Pre-training simulation	32 (45.1)	71	Post-training simulation	45 (63.4)	-	-
				Participants who responded “Strongly agree” to: I would help a client get any contraceptive method they want, even if I think they should be using something else	71	Pre-training simulation	41 (57.8)	71	Post-training simulation	50 (70.4)	-	-
				Participants who responded “Strongly agree” to: Contraceptive services are an important part of healthcare	71	Pre-training simulation	53 (74.7)	71	Post-training simulation	55 (77.5)	-	-
O’Laughlin, 2020	Pregnancy Reasonably Excluded Guide (PREG) Evaluation	981 women aged 18 to 50 years who were seen in a primary care gynecology clinic for IUC or implant placement	LARC	Same-day placement of LARC	1,012 visits	Before PREG implementation^*^	594 (58.7)	1,012 visits	After PREG implementation	974 (96.2)	-	-
Harper, 2020	Contraceptive training intervention	Clinic staff	IUD	Require 1 visit to place device	3,216	Baseline	836 (26)	2,126	Month 3	744 (35)	OR (Adj.)^†^	1.6 (1.4–1.8)
			Implant	Require 1 visit to place device		Baseline	996 (31)		Month 3	871 (41)	OR (Adj.)^†^	1.5 (1.3–1.7)
		Training intervention- Overall	IUD	Require 1 visit to place device	4,728	-	-	-	-	-	OR (Adj.)^†^	2.0 (1.3–2.8)
			Implant	Require 1 visit to place device	4,693	-	-	-	-	-	OR (Adj.)^†^	1.9 (1.2–2.9)
		Primary care	IUD	Require 1 visit to place device	4,728	-	-	-	-	-	OR (Adj.)^†^	0.7 (0.4–1.5)
			Implant	Require 1 visit to place device	4,693	-	-	-	-	-	OR (Adj.)^†^	1.0 (0.5–1.9)
		Family planning clinic	IUD	Require 1 visit to place device	4,728	-	-	-	-	-	OR (Adj.)^†^	1.5 (0.8–2.9)
			Implant	Require 1 visit to place device	4,693	-	-	-	-	-	OR (Adj.)^†^	2.0 (1.0–3.8)
		Hospital	IUD	Require 1 visit to place device	4,728	-	-	-	-	-	OR (Adj.)^†^	2.0 (1.0–4.2)
			Implant	Require 1 visit to place device	4,693	-	-	-	-	-	OR (Adj.)^†^	2.3 (1.1–4.6)
		Other	IUD	Require 1 visit to place device	4,728	-	-	-	-	-	OR (Adj.)^†^	1.3 (0.7–2.5)
			Implant	Require 1 visit to place device	4,693	-	-	-	-	-	OR (Adj.)^†^	1.6 (0.8–3.0)
Buckel, 2019	Complete CHOICE	Overall	Hormonal IUD	Same-day placement of LARC	502	Enhanced care	10 (2)	506	Complete CHOICE	40 (7.9)	-	p-value < 0.01
			Copper IUD	Same-day placement of LARC		Enhanced care	2 (0.4)		Complete CHOICE	11 (2.2)	-	p-value < 0.01
			Implant	Same-day placement of LARC		Enhanced care	17 (3.4)		Complete CHOICE	110 (21.7)	-	p-value < 0.01
DeBoer, 2018	Quality improvement project	Overall	LARC	Same-day placement of LARC	70	2007 criteria	0 (0)	15	2017 criteria	8 (53.3)	-	-
Biggs, 2015; Natavio, 2018	California Family PACT	Providers and staff at Family PACT practice sites	IUD (any type)	Requires 1 visit	413	2011 survey	174 (42.0)	-	-	-	-	-
			Implant	Requires 1 visit	221	2011 survey	118 (53.0)	-	-	-	-	-
			LARC	Provide same-day placement	-	-	-	102	2016 survey	16 (16.0)	-	-
				Provide conditional same-day placement	-	-	-	102	2016 survey	14 (14.0)	-	-
				Unsure about same-day placement	-	-	-	102	2016 survey	(6.0)	-	-
Loyola Briceno, 2017	Performance Measure Learning Collaborative	PMLC county health department	LARC	Proportion of LARC placements occurring on the same-day as patient’s initial consultation	1	November 2015	(36)	1	May 2016	(50)	-	-
Romero, 2015/2017	National demonstration project providing technical assistance and training	Health centres partnered with the CDC and Office of Adolescent Health teen pregnancy prevention community-wide initiative	IUD	Provide same-day placement	51	Baseline (2011)	6 (12.5)	48	Year 2 (2012)	25 (52)	-	-
									Year 3 (2013)	28 (58)	-	-

*Abbreviations*: *Adj* Adjusted, *CAP* Contraceptive Access Program, *CDC* Centers of Disease Control and Prevention, *DelCAN* Delaware Contraceptive Access Now, *DID* Difference-in-difference, *FPE* Family Planning Elevated, *IUC* Intrauterine contraceptive, *IUD* Intrauterine device, *LARC* Long-acting reversible contraception, *OR* Odds ratio, *PMLC* Performance Measure Learning Collaborative, *PREG* Pregnancy Reasonably Excluded Guide

*If traditional criteria, current menses, or presence of a current IUC or implant had been used to exclude pregnancy, only 594 (58.7%) would have qualified for a same-day procedure

†Adjusted for provider type, training year, region; Planned Parenthood reference

‡A simulated comparison group of women who were receiving care through the Missouri Title X program and modeled the contraception and pregnancy outcomes that would have occurred in the absence of the Contraceptive CHOICE Project

Publications for five programs did not provide quantitative results pre- and post-program implementation (Appendix Table C2 in Supplementary Material) [30, 32, 37, 42, 53, 54]. A quality improvement project utilized counseling and quick-start for contraceptive methods; however, due to insurance requirements in order to secure no-cost for the patient, none of the 14 patients who opted for the implant could quick-start the method [32]. Statewide initiatives to grant funding to Title X and non-Title X clinics in Iowa and Colorado resulted in 49% (IUD) and 59% (implant) of clinics only requiring 1 visit for placement [30]. Federal funding provided to Texas clinics participating in the Healthy Texas Women (HTW), Title X, and Family Planning Program (FPP) initiatives reported same-day placement rates ranging from 22% to 79% for IUDs and 22% to 83% for implants [54]. 

While not a newly implemented program, one study evaluated LARC uptake immediately following surgical abortion at three Planned Parenthood League of Massachusetts clinics after the implementation of the contraceptive coverage mandate of the Affordable Care Act (ACA) in 2012 [50]. In this setting, health counselors offered the option of post-abortion LARC prior to the procedure, all abortion providers had the capacity to place post-abortion LARC, and clinics were well-stocked with devices. Rates of immediate post-abortion LARC placement rose from 18.0% in 2012 to a peak of 29.0% in 2015, before dropping slightly to 28.3% in 2016. Placement of both the IUD and implant rose over the study period, with rates rising 4.3% for IUDs and 8.0% for implants between 2012 and 2016.

### Economic impact of same-day LARC placement

Two studies reported on the economic impact of same-day LARC placements. Using a base case of a 16-year old patient presenting for care and desiring LARC, a cost-minimization analysis from the Medicaid perspective found that there was cost savings for providing same-day LARC placements to adolescents [55]. Assumptions included in the analysis were that the chosen LARC method would be available for same-day placement, if a patient did not return for LARC, she would not use another form of contraception, no-cost pregnancy termination (as there is no Medicaid coverage for this in most states), and pregnancy outcomes were term delivery, preterm delivery, miscarriage, or termination. Additionally, twin pregnancies, pregnancy complications (outside cesarean delivery), and long-term health care costs for children were not included. Probabilities and costs of each outcome, along with the probability of continuing LARC for 1 year were derived from the literature. The resulting one-year net cost of providing same-day LARC per adolescent was $2,016 (2017 United States dollar [USD]), with estimated cost savings of $2,117 USD per year per LARC placement when compared to requiring a second visit.

A cost-effectiveness analysis evaluated the potential cost savings associated with scaling-up of the Contraceptive CHOICE Project in Missouri to all statewide Missouri Medicaid beneficiaries [46]. A simulated comparison group was created to demonstrate cost savings if the CHOICE Project model were scaled up, using contraceptive costs, discontinuation rates, and unintended pregnancy-related events from the CHOICE Project. In their model, if 20% of the women who are 18–45 years of age and currently enrolled as Medicaid beneficiaries in the state used LARC, the cost savings would be approximately $80 million (2013 USD); if 40% used LARC, these savings would increase to approximately $157 million annually.

### Key recommendations identified in the literature

Table 5 presents a comprehensive overview of barriers, facilitators, and recommendations from the authors of publications included in this review, regarding programs aimed at increasing access to same-day LARC. These recommendations have been categorized into domains, including health system strengthening, program modifications, clinical protocols, research, service expansion, and advocacy. Actionable insights for each domain were synthesized from the recommendations and are as follows:

**Table 5:** Key findings and recommendations to increase same-day access to LARCs

Domain	Identified barrier	Recommendation	Author, year
Health system strengthening(12 recommendations)	Barriers		
	Lack of integrated approaches	Develop health-system wide approaches to ensure single-visit LARC.	Stuart, 2023
	Mixed adherence among providers to practice guideline recommendations for same-day LARC provision	While designing interventions to improve LARC access, use information on identified implementation barriers to guide recommended practices.	Judge-Golden, 2020
	Lack of tailor-made interventions that suit individual patient requirements (e.g., depressed patients)	Offering same-day LARC services may be particularly critical for depressed patients, who may need contraceptive methods that do not require frequent or self-administration. SRH clinicians can anticipate the issues related to understanding, adherence, and communication with partner to best guide a young woman with depression in her choice of contraceptive methods and support its successful use.	Katz-Wise, 2020
	Health system administrative and financial barriers affect same-day LARC placement	Future research might include questions about the presence of specific financial, administrative, or other barriers to LARC provision in outpatient clinical practice.	Kelly, 2017
	Inability to provide same-day IUD placements for teens is a significant barrier	Regular clinic assessments to examine health center practices and capacity in the provision of reproductive healthcare for adolescents with the purpose to identify opportunities for health center improvement.^*^	Romero, 2015
	Accessibility and quality of reproductive health services for adolescents need improvement	Focus on strategies to build the capacity of health center partners to ensure that the evidence based clinical practices are implemented, thus enhancing the overall accessibility and quality of reproductive health services for adolescents in the initiative.	Romero, 2015
	Facilitators		
	Capacity building of clinic staff along with physicians	Involve clinic staff in the educational efforts (teaching, training, and proctoring) directed to physicians. Focus on provider training, particularly for the contraceptive implant, and systems and policy changes that will improve availability of same-day IUD placement and adequate provider reimbursement.	Tepper, 2020, Luchowski, 2014
	Training programs for primary care providers	As ‘Title X’ funding is restricted from reaching many reproductive health specialist providers, training programs can help to advance contraceptive care and build primary provider capacity to offer patients a variety of contraceptive methods in diverse clinic settings. Effective program diffusion in a nationwide scale-up is important.	Harper, 2020
	Incentivizing same-day LARC through effective reimbursement strategies	Efforts for creating and implementing reimbursement strategies that incentivize same-day LARC placement are feasible and should be undertaken in all clinical settings.	Wilkinson, 2019
	Strengthening same-day LARC placement coverage through public health clinics	Public health clinics are in an excellent position to address unintended pregnancy rates by increasing access to all contraceptive methods, especially LARCs, the recommended first-line contraceptive method.	DeBoer, 2018
	Transferability of evidence from emergency to non-emergency settings	Z-CAN’s design and implementation could be refined and adapted in other non-emergent settings, in which increased access to contraception could improve health outcomes.	Lathrop, 2018
	Using performance measures in the context of a learning collaborative may be a useful strategy for other programs (e.g., Federally Qualified Health Centers, Medicaid, private health plans)	Expanded use of performance measures may help increase access to contraceptive care and achieve national goals for family planning by providing other programs with an effective strategy to contraceptive care.	Loyola Briceno, 2017
Program modifications (10 recommendations)	Barriers		
	Non-availability of IUD or implant	Program modifications that allow all HTW providers to purchase and stock IUDs and implants in advance of patients’ request would improve timely initiation of these methods for those who want them.	Vohra-Gupta, 2022
	Annual certification requirement related to provision and promotion of abortion care affects uptake negatively	The removal of the annual certification requirement that HTW providers do not provide or promote abortion care could increase other family planning providers’ participation in the HTW program. This would affect more than those affiliated with Planned Parenthood, and it could reduce information barriers for patients who do not want to continue their pregnancies.	Vohra-Gupta, 2022
	Lack of Continued Medical Education	Medical and legal education regarding IUD provision to adolescents should be provided across all levels of clinic staff to reduce barriers to care.Continuing education strongly predicted whether obstetrician–gynecologists inserted implants and was also associated with other practices that encourage LARC use.Concurrently, improving providers' understanding of and adherence to current professional guidelines, including the benefits of different kinds of LARCs for a wider array of clients, will hopefully optimize the mainstreaming of LARC placement during the client's first visit.	Lim, 2020, Luchowski, 2014, Biggs, 2013
	LARC onsite availability	Only a minority of Family PACT practices said that they had LARC devices available onsite, which imposes a substantial restriction to access for women who are entitled to have access without cost under this program. Other states developing same-day placement programs should be aware of this challenge when trying to address availability.	Natavio, 2018
	Cost barrier	The potential cost savings of scaling up a model of care similar to the CHOICE Project are large. State Medicaid programs that are looking at budget priorities should focus on the removal of barriers to all contraceptive methods.	Madden, 2018
	Facilitators		
	Robust provider networks facilitate uptake and help address cost barrier	Ensuring a robust provider network will involve efforts to sustain those organizations that see a large volume of family planning patients, as well as strengthening training and education for providers at small-volume sites.Robust provider networks should be encouraged to ensure adequate reimbursement and offering funding supports to facilitate patient enrolment and use of program services.	Vohra-Gupta, 2022
	Integrating telemedicine and ensuring patient privacy and confidentiality	Clinics may benefit from established protocols that better integrate telemedicine workflows with LARC, which may vary depending on a patient’s readiness for LARC and whether the practice offers same-day placement.Further interventions are needed to improve patient privacy, such as chat functions to offer safety check-ins for patients if they unable to verbally confirm their safety and/or confidentiality.	Song, 2022
	Utilizing a “Buy and Bill” model to expand access to LARC devices	Advocacy efforts with ACA plan administrators for a “Buy and Bill” model to address LARC stocking issues and expand access to LARC devices could encourage the consideration of cost-effectiveness of full-spectrum contraceptive coverage.	Davis, 2020
	Maintain clinic inventory of LARCs	Maintaining clinic LARC inventory to facilitate same-day placement when desired.All methods of contraception, regardless of the upfront cost, should be readily available to all women to allow them to obtain their preferred method.	Buckel, 2019
	Increasing appointment length	Addressing clinic flow to allow for longer appointments would facilitate same-day placement when desired.	Buckel, 2019
Clinical protocols (2 recommendations)	Barrier		
	Pregnancy checklist criterion alone are barriers	Α shared decision-making conversation on the risks and benefits of immediate levonorgestrel-releasing IUS (LNG-IUS) placement based on the pregnancy checklist criteria alone. They should, rather, combine clinical decision making and liberal use of EC with immediate provision of LNG-IUS.	Castaño, 2020
	Facilitator		
	Tools that help consistent and objective documentation of pregnancy status (PREG checklist) decrease unintended pregnancy rates	PREG presents a structured opportunity for shared decision making regarding preprocedural pregnancy testing and its continued implementation in other settings will aid in time- and cost-efficient healthcare for reproductive-aged women.	O’Laughlin, 2020
Research (11 recommendations)	Barrier		
	Bias in service provision is a potential barrier	Investigate presence of bias at multiple levels of each clinical encounter	Serpico, 2020
	Facilitators		
	Strengthening comprehensive contraceptive access	Elucidate efficient and effective interventions and implementation practices around IUD training (counseling, referral services) where IUD services are not available.	Reeves, 2023
	Assessment of patient and provider factors affecting same-day LARC	Examine how patient preference or medical necessity may influence the rate of same-day LARCs.	McColl, 2022
	Understanding of barriers facilitates improvement efforts	Assess the extent to which different barriers limit IUD uptake to guide improvement efforts.	Lim, 2020
	Feasibility assessment of implemented models	Explore the feasibility of instituting interventions such as incentive payments, reconfiguring reimbursement structures, or pursuing bulk purchasing strategies to promote and facilitate efforts to increase same-day LARC availability and improve access to contraceptive care.	Wilkinson, 2019
	Pediatric primary care offices can offer quality comprehensive adolescent SRH services	More research and health policy advocacy are needed to support private practices in overcoming current external barriers.	Landgraf, 2019
	Improve the implementation of evidence based clinical practices.	Efforts to identify the barriers and facilitators for this improvement process can inform health centers of opportunities to enhance their capacity and ensure that evidence-based clinical practices are being implemented.	Romero, 2017
	Dissemination of results of successful implementation strategies	Identifying approaches used by practices that have been successful in implementing same-day placement protocols could be used to develop and implement strategies among those healthcare providers facing the greatest challenges.	Biggs, 2015
	Barriers and facilitators to implementation of the evidence-based clinical practices need to be understood	Identify the barriers and facilitators to implementation of the evidence-based clinical practices to facilitate improvement efforts e.g., support from health center leadership and providers, communication between leadership and staff overseeing implementation, the use of data for CQI, and attitudes and beliefs among providers to address the reproductive health needs of adolescents.	Romero, 2015
	Understanding mechanisms of impact	Supporting health center capacity and systems needs to ensure consistent and quality implementation of evidence-based clinical practices will improve adolescent access and use of reproductive health services, ultimately decreasing teen pregnancy and birth rates. Evaluate community-wide initiatives to further understand the impact of how community partnerships and initiatives are facilitating adolescent access to and use of reproductive health services.	Romero, 2015
	Research should allow for studies of patient factors influencing contraceptive choice to analyze IUD and implant uptake as separate outcomes	While clinicians and researchers often group LARC methods based on their similarly high effectiveness, this study demonstrates that women selecting one or the other LARC device vary significantly along demographic and social lines and may differ in their contraceptive decision-making. Further investigation is required to understand how women choose their post-abortion contraceptive method and how their personal histories impact that choice.	Roe, 2019
Implementation research (2 recommendations)	Barrier		
	Interventions focusing on counseling alone were insufficient in increasing same-day LARC	Interventions must also address other barriers to contraceptive access including provider training, on-the-shelf LARC, and cost to truly increase access for patients.	Buckel, 2019
	Facilitator		
	Low cost facilitated simulation, debriefing, and action planning helps counseling	Highly realistic family planning simulations can be used to assess implementation fidelity in a way that is acceptable, non-threatening and effective.	Cohen, 2022
Advocacy (3 recommendations)	Barrier		
	Inequities in health-care provision	Expand eligibility and advocate for same-day placement of LARCs within public health system.	Lathrop, 2018
	Facilitators		
	Advocacy for single-visit LARC	Providing single-visit LARC is evidence-based, patient-centered, and reduces healthcare costs.	Stuart, 2023
	Advocacy for policy change in service provision	Identify capacity-related issues and ways availability can be improved. Education has certainly been useful to improve same-day LARC uptake, but different reimbursement scales may motivate change more effectively. These changes in financial incentives may improve sustainability and demonstrate that payers value and prioritize high quality contraceptive services. However, this must be done with policies that ensure the availability of all contraceptive methods on site, including LARCs.Pediatric primary care offices can offer quality comprehensive adolescent SRH services	Natavio, 2018, Landgraf, 2019

*Abbreviations*: *ACA* Affordable Care Act, *CME* Continuing Medical Education, *CQI* Continuous Quality Improvement, *EC* Emergency contraception, *HTW* Healthy Texas Women, *IUD* Intrauterine device, *LARC* Long-acting reversible contraception, *LNG-IUS* Levonorgestrel-intrauterine system, *PACT* Planning, Access, Care, and Treatment, *PREG* Pregnancy Reasonably Excluded Guide, *SRH* Sexual reproductive health, *STI* Sexually transmitted infection, *Z-CAN* Zika Contraceptive Access Network

*Specifically, information collected was used to identify where targeted improvement efforts were needed for adherence to evidence-based clinical practices, including access, processes for the delivery of care, utilization of cost, confidentiality, supportive infrastructure, and the healthcare delivery environment

#### Health system strengthening

Twelve recommendations focused on strengthening the health system, from which three main actionable insights were drawn. First, implementation of system-wide, integrated training interventions aimed at improving the ability of clinic staff to place LARC devices should be a priority, as this increases access to same-day LARC services [39, 45, 53]. Second, where a lack of integrated approaches and mixed adherence to practice guidelines were identified, barriers to same-day LARC uptake remained [40, 52]. Cost and administrative hurdles were shown to impede same-day LARC uptake across outpatient practices. Incentivizing effective reimbursement strategies, such as a single, uniform reimbursement structure across insurers (e.g., Medicaid) as a medical benefit, was recommended as a way to reduce costs associated with contraceptive care, introduce substantial savings, and address administrative barriers that impede same-day LARC uptake across outpatient practices [41, 55]. Lastly, ensuring the transfer of evidence between states and programs will help increase access and quality of reproductive health services nationwide [28]. This can be achieved through expansion and standardization of performance measures in contraceptive care and testing the adoption of prior successful approaches in emergency to non-emergency settings [42]. 

#### Program modifications

Ten recommendations centered around the modification of existing programs and infrastructure, and focused on addressing dimensions of access, availability, cost, and insurance. Creation and expansion of robust provider networks is needed and will allow for persistent cost and insurance barriers to be addressed. In particular, provision of financial support that facilitates stocking LARC devices in advance of placement was highlighted as a way to overcome this access barrier as many clinicians cannot afford to order devices prior to ensuring pre-authorization of insurance coverage at the time the device is requested [27, 35, 54]. A buy-and-bill program added to regular contraceptive care in Hawai’i provided financial support and reduced upfront cost to patients [37]. Addressing this barrier, along with increasing appointment length and ensuring patient privacy and confidentiality in contraceptive programs, had a positive effect and facilitated contraceptive care [34, 35]. 

#### Clinical protocols

Identified by two recommendations, the development and implementation of more effective tools to determine LARC eligibility are needed to reduce the reliance on evaluating pregnancy status alone prior to LARC provision. A successful test of the Pregnancy Reasonably Excluded Guide (PREG) checklist showed that consistent and objective documentation of pregnancy status decreased unintended pregnancy rates; [48] basing the immediate placement of an IUD on traditional pregnancy checklists that require normal menses alone was a barrier to access [36]. A focus on shared decision-making regarding LARC placement and addressing bias in service provision will also help to remove clinical barriers and improve access to same-day LARC.

#### Research

Eleven recommendations centered on approaches to conducting future research to facilitate access to same-day LARC services. From these, four actions were identified to continue addressing barriers in contraceptive care. First, more work is needed to further improve the implementation of evidence-based clinical practices to ensure consistency in service provision [28, 29]. Second, the development and implementation of efficient and effective programs for LARC training were proposed [26, 49]. Third, steps to understand patient perspectives and preferences were highlighted in order to help mitigate any potential bias in reproductive service provision [28, 43, 47, 51]. Lastly, studies analyzing IUD and implant uptake should consider these as separate outcomes (as opposed to grouping all LARC devices together) to allow for the evaluation of factors that influence contraceptive choice [50]. 

#### Implementation research

Two recommendations focused on improving program implementation. Clinic-wide, low-cost family planning simulations have the ability to engage staff in a training program and can increase counseling intervention effectiveness [31]. Adding cost support and provider training to a program alongside standard patient-centered counseling was shown to have a greater increase in same-day LARC uptake as compared to a program that focused solely on counselling [35]. Hence, incorporating a multi-faceted approach to contraceptive programs will have the opportunity of greater impact and ability to overcome current barriers to same-day LARC uptake.

#### Advocacy

Three recommendations on advocacy to address system-wide concerns were highlighted in the literature. Facilitation of policy changes is required to help motivate change in service provision and can be accomplished by continuously addressing eligibility concerns and inequities within the public health system [27, 32]. Advocating for increased on-site availability of LARC methods along with the introduction of financial incentives and different reimbursement scales will address these inequities and expand same-day LARC eligibility [27, 42, 52]. 

## Discussion

Same-day LARC placement is recommended by the WHO, ACOG, and CDC as an effective best practice that improves access to contraception; still, same-day LARC utilization remains low across the US with some studies in this review reporting no same-day LARC placements prior to program implementation [5, 15, 16, 19]. Streamlining the process for patients to receive LARC on the same day they receive contraceptive counseling, highlighting strategies to enhance access to same-day LARC methods, and addressing associated challenges are important steps in improving contraceptive care in the US [17]. As a result, several different programs have been implemented across the US aimed at increasing the rate of same-day LARC placements. Successful programs showed an increase in the number of same-day LARC placements through improved access and the removal of barriers to contraceptive care. Substantial cost savings were also estimated to be associated with same-day LARC placement. To our knowledge, this is the first literature review to synthesize published evidence on the effectiveness of programs aimed at increasing same-day LARC placement, and to identify the factors that remove barriers or that are associated with program success.

From the 30 articles identified, spanning 15 programs and 21 states, a consistent trend of improved rates of same-day LARC placements were documented, despite variability in settings and features of the programs. For example, the DelCAN program, which supplied direct financial resources, improved LARC device availability, and provided training and administrative support to providers between 2015 and 2019, showed a substantial increase in the same-day placement of LARC devices (25.6% increase for implants and 17.4% for IUDs) [47]. Similarly, the Complete CHOICE program, which focused on providing contraceptive counseling, healthcare provider training, in-clinic stocking of LARCs, and no-cost provision of LARCs to uninsured individuals, resulted in 18% more implant placements in the Complete CHOICE group than in the Enhanced Care comparison group [35]. In an emergency setting, the Zika Contraceptive Access Network (Z-CAN) program combated the effects of Zika virus through increased access to contraceptives in Puerto Rico by increasing provider training, availability of LARC devices, funding for clinics, and patient counseling. This program resulted in 33.9%, 23.8%, and 9.8% of women receiving a hormonal IUD, implant, or copper IUD, respectively at their initial visit [42]. Prior to the implementation of Z-CAN in 2016, only 3.9% of 3,059 women surveyed were using LARC, of which, all were IUDs [57]. While most programs aiming to address cost-related barriers to LARC access covered clinic costs upfront, or offered subsidies to individuals seeking LARC placement, the PMLC helped clinics streamline their reimbursement processes, and advocated for encouraging individuals to register for either public or commercial insurance to avoid paying for LARC out-of-pocket [44]. 

Programs that focused on the implementation of evidence-based clinical practices also showed an increase in the uptake of same-day LARCs. Limiting the requirement for STI testing to high-risk women, and removing cervical cancer screening as a requirement prior to LARC placement, showed a 53% increase in the number of same-day placements when compared to programs implemented using the previous criteria [38]. When these evidence-based clinical practices were focused on supporting provision of youth-friendly healthcare throughout the US, a year-over-year increase was seen in the number of clinics able to provide same-day LARC placement to teens (year 1: 39.5%, year 2: 6%).

Single-factor programs such as those evaluated by O’Laughlin et al., 2020 (PREG evaluation) and Harper et al., 2020 (healthcare provider training) were also associated with increases in same-day LARC placement, indicating that programs aimed to address a specific barrier to same-day LARC placement can also lead to positive impacts to contraceptive care. Implementation of the PREG checklist resulted in 37.5% more women receiving same-day LARC placements than if they were evaluated using traditional criteria (i.e., pregnancy testing via blood hCG levels) or current menses alone [48]. In the study by Harper et al., 2020, clinic staff attended a one-day course that used a team approach to promote clinical practice change, enhance the skills of the healthcare team to allow task-sharing, and streamline clinic requirements to reduce multiple patient visits. This contraceptive training intervention led to an overall increased clinic ability to provide same-day implant (OR:1.9, 95% CI: 1.2–2.9) and IUD (OR: 2.0, 95% CI: 1.3–2.8) placements [39]. 

Transitioning to same‑day placement may help decrease product abandonment—a scenario in which a device is procured and approved for a patient through insurance, yet the patient fails to return for the subsequent placement appointment. However, maintaining an inventory of LARC devices for same‑day placement may be challenging for some clinics due to the upfront costs associated with stocking these products [13, 46]. Smaller health systems or single clinics may not have the capability or available funding to provide comprehensive same-day LARC programs that may be available in larger, more extensive networks [41, 52]. Considerations should be made, however, based on evidence showing that although LARC devices require a substantial initial acquisition cost, these expenses are rapidly offset by reductions in pregnancy‑related healthcare expenditures [58]. 

While targeted same-day placement programs generally showed increased LARC placement rates, the extent of improvement observed between each program varied thus potentially indicating that residual barriers still exist. Relieving health system barriers including potential biases in service provision may increase the effectiveness of any given intervention.

Additionally, even though improvements were observed in single facet programs such as PREG, this type of program did not address the other barriers to LARC placement faced by both clinicians and patients. Buckel et al., 2019 showed that interventions focused on counseling alone were insufficient in increasing same-day LARC and recommended that interventions also consider provider training, on-the-shelf LARC, and potential cost offsets [35]. It is worth noting that even if key barriers are reduced, preferences and other individual factors may preclude real-world utilization from approaching this level [59]. This can be highlighted through utilization rates in other countries, with LARC use estimated at approximately 10% in Canada and 20% in Sweden [60, 61]. 

The two studies that evaluated the economic impact of same-day LARC placement in the US provided evidence of positive economic impacts. Madden et al. report the potential for substantial cost savings and utilization of same-day LARC placement across multiple utilization scenarios [46]. The available economic research provides supporting evidence to ongoing cost savings across the range of possible LARC placement rates. While positive economic impacts were described, the evidence was sparse and evaluated Medicaid populations only, therefore lacking generalizability. Furthermore, due to the fragmented nature of the US healthcare system, the costs to implement same-day LARC may be accrued by a different organization than the one that saves costs due to reduced unintended pregnancies or abortions, or on a longer timeframe than the one used by the included cost-effectiveness analysis. While both included economic analyses evaluated same-day LARC within a state Medicaid system, the results of these analyses should be interpreted with caution as they may not be reflective of situations and systems outside of Medicaid. More research is needed to fully elucidate the cost-effectiveness of same-day LARC provision in the US.

This literature review was designed to capture contemporary evidence from 2012 onwards, which allowed for capturing changes to recommendations by organizations such as ACOG and WHO. The study also captured the introduction of the contraceptive coverage mandate of the ACA, which resulted in increased LARC use throughout the US due to required coverage through insurance plans to reduce out-of-pocket costs [62]. However, as noted by studies included in this review, ensuring access, affordability and adequate reimbursement for all patients to receive same-day LARC placement remains a concern [40, 52, 54]. Additionally, since the search was conducted, further recommendations for clinical practice have been published. One example is from the CDC, outlining how to be reasonably certain that a patient is not pregnant prior to initiating contraceptives [63]. Clinical practice recommendations such as this aim to address clinical protocol barriers and represent action taken on identified barriers to same-day LARC captured in this review.

Advocacy and calls-to-action among the included literature highlighted inequities in health care provision, the benefits of same-day LARC placement, and the positive policy changes in service provision [32, 42, 52]. The removal of identified system-wide barriers such as mixed adherence to practice guidelines, administrative barriers, and high cost should also continue to be advocated for as shown by the success of the included same-day LARC placement programs. Additionally, any future changes in financial incentives or loss of funding for same-day LARC placement will further increase barriers to access. Actionable insights identified from our review are in line with findings from other publications investigating the delivery of contraceptive care for women of reproductive age. These studies identified the need to implement interventions addressing provider bias and knowledge, clinic operations and policies, confidentiality concerns, and insurance reimbursement to improve access [64–67]. Previous studies have also reported a lack of counselling and LARC placement skills in providers, and absence of hospital protocols for related interventions, as barriers to LARC uptake. Knowledge deficits and misconceptions among adolescents and their healthcare providers were identified as barriers for adolescents in these publications [7, 8]. Acting on the recommendations identified in the literature to improve access to same-day LARC, such as those considered to strengthen the health system, may also result in increased overall utilization. While the two-visit approach may also accomplish this and be preferred by some patients (e.g., need time to think about it) and providers (e.g., insurance pre-approval and receiving more pay for two visits) even with the recommendation for same-day placements by the ACOG and CDC, increasing access to LARC overall reduces unintended pregnancy rates and improves health outcomes among reproductive age women.

Although concerns about LARC discontinuation and placement‑related pain are not consistently cited as barriers to same‑day placement, they may indirectly influence provider and patient decision‑making [68, 69]. Reported discontinuation rates range from approximately 11% to more than 50% in the first year, leading some clinicians to prefer a two‑visit model to allow additional time for counseling and confirmation of method suitability [14, 30, 70, 71]. From the patient perspective, anticipated difficulty obtaining device removal and concerns about early discontinuation may deter initial LARC uptake [72, 73]. Furthermore, perceived pain associated with LARC insertion may represent an additional patient-level barrier, particularly in the context of same-day placement [69]. Comprehensive pre‑procedure counseling and expectation‑setting may help mitigate these concerns.

Rigorous quality assessments were undertaken to ensure correct interpretation of included data. Nonetheless, some limitations should be noted. First, most of the identified evidence relies on self-reported data (e.g., provider phone interviews or surveys) which is prone to information bias away from the null; however, evidence from studies relying on other types of data was in alignment [74], which helped mitigate this concern. Second, access to and utilization of LARCs was the primary outcome in most studies, with same-day placement reported as a secondary outcome. However, availability of authors’ conclusions on the barriers to same-day LARC placement lend confidence that there is no resulting impact on our conclusions. Next, same-day LARC placement outcomes specific to sub-populations of women desiring use, or current users of LARCs, were not readily available; thus, insights tailored to such a population could not be derived. Another important limitation relates to the generalizability of the study findings. Due to the focus on the US health system, identified benefits and barriers to same-day LARC placement may not be generalizable to other countries. Programs that were implemented in one state alone (e.g., CHOICE) or in an emergency setting (e.g., Z-CAN) may not be generalizable. While many other programs (including some included in this review) styled their approach after the CHOICE project, state policy and healthcare infrastructure may impede close replication outside of Missouri. In an emergency setting such as Z-CAN, the observed impact of the contraceptive program on same-day LARC placement rates will be an overestimation due to many women likely being driven to seek contraceptive care due to the external emergency, as opposed to improved access alone. Additionally, many of the recommendations are from the perspective of or apply to larger health systems and clinics, which may reduce generalizability to smaller, underserved settings. Also, while the review captured publications from 2012 onwards, some of the evaluated programs may have predated the introduction of the contraceptive coverage mandate of the ACA in August 2012, particularly in consideration of lags in implementation. Lastly, there are no standardized procedure codes to identify same-day LARC placements in EMR data, so there were variations in how “same-day”, or “single-visit” were defined among studies. This could result in misclassification bias as same-day LARC uptake may be reported as being higher than it is. Further research into the best approach to identifying and recording same-day LARC placements in EMR is warranted.

## Conclusion

Our review has shown that programs aimed at increasing same-day LARC placement in the US have been largely effective and may be cost-saving. Implementing changes such as providing education on LARC devices, implementing practical policy initiatives for access centered on patient and healthcare provider contraception preferences, and patient-centered counseling approaches along with hands-on LARC placement training to healthcare providers, streamlining of pregnancy and screening requirements, providing financial assistance, and supporting LARC availability in-clinic are strategies shown to be effective in removing barriers to same-day LARC placement. Opportunities to ensure same-day contraceptive access include promoting user and provider awareness, extending funding, decreasing administrative hurdles, and building trust in contraceptive care. A wider implementation of these strategies will lead to improved contraceptive access in the context of increased use of LARC devices in the US.

## Data Availability

The datasets and protocol generated for this study are available from the corresponding author upon reasonable request.

## Abbreviations

ACA: Affordable Care Act
ACOG: American College of Obstetricians and Gynecologists
CDC: Centers for Disease Control and Prevention
hCG: Human chorionic gonadotropin
IUDs: Intrauterine devices
LARC: Long-Acting Reversible Contraceptive
MMAT: Mixed Methods Appraisal Tool
DelCAN: Delaware Contraceptive Access Now
PICOS: Population, Intervention, Comparator, Outcomes, and Study
PREG: Pregnancy Reasonably Excluded Guide
PRISMA: Preferred Reporting Items for Systematic reviews and Meta-Analyses
STI: Sexually transmitted infection
SLR: Systematic literature review
USD: United States dollar
WHO: World Health Organization
Z-CAN: Zika Contraceptive Access Network
